# Multiple Mechanisms of NOTCH1 Activation in Chronic Lymphocytic Leukemia: NOTCH1 Mutations and Beyond

**DOI:** 10.3390/cancers14122997

**Published:** 2022-06-17

**Authors:** Federico Pozzo, Tamara Bittolo, Erika Tissino, Antonella Zucchetto, Riccardo Bomben, Laura Polcik, Svenja Dannewitz Prosseda, Tanja Nicole Hartmann, Valter Gattei

**Affiliations:** 1 Clinical and Experimental Onco-Hematology Unit, Centro di Riferimento Oncologico di Aviano (CRO), IRCCS, 33081 Aviano, Italy; tamara.bittolo@cro.it (T.B.); etissino@cro.it (E.T.); zucchetto.soecs@cro.it (A.Z.); rbomben@cro.it (R.B.); vgattei@cro.it (V.G.); 2 Faculty of Biology, University of Freiburg, 79106 Freiburg, Germany; laura.polcik@uniklinik-freiburg.de; 3 Department of Internal Medicine I, Faculty of Medicine and Medical Center, University of Freiburg, 79106 Freiburg, Germany; svenja.dannewitz@uniklinik-freiburg.de (S.D.P.); tanja.hartmann@uniklinik-freiburg.de (T.N.H.)

**Keywords:** NOTCH1, FBXW7, chronic lymphocytic leukemia, gene mutations

## Abstract

**Simple Summary:**

Mutations of the *NOTCH1* gene are a validated prognostic marker in chronic lymphocytic leukemia and a potential predictive marker for anti-CD20-based therapies. At present, the most frequent pathological alteration of the NOTCH1 gene is due to somatic genetic mutations, which have a multifaceted functional impact. However, beside *NOTCH1* mutations, other factors may lead to activation of the NOTCH1 pathway, and these include mutations of *FBXW7*, *MED12*, *SPEN*, *SF3B1* as well as other B-cell pathways. Understanding the preferential strategies though which CLL cells hijack NOTCH1 signaling may present important clues for designing targeted treatment strategies for the management of CLL.

**Abstract:**

The Notch signaling pathway plays a fundamental role for the terminal differentiation of multiple cell types, including B and T lymphocytes. The Notch receptors are transmembrane proteins that, upon ligand engagement, undergo multiple processing steps that ultimately release their intracytoplasmic portion. The activated protein ultimately operates as a nuclear transcriptional co-factor, whose stability is finely regulated. The Notch pathway has gained growing attention in chronic lymphocytic leukemia (CLL) because of the high rate of somatic mutations of the *NOTCH1* gene. In CLL, NOTCH1 mutations represent a validated prognostic marker and a potential predictive marker for anti-CD20-based therapies, as pathological alterations of the Notch pathway can provide significant growth and survival advantage to neoplastic clone. However, beside *NOTCH1* mutation, other events have been demonstrated to perturb the Notch pathway, namely somatic mutations of upstream, or even apparently unrelated, proteins such as *FBXW7*, *MED12*, *SPEN*, *SF3B1*, as well as physiological signals from other pathways such as the B-cell receptor. Here we review these mechanisms of activation of the NOTCH1 pathway in the context of CLL; the resulting picture highlights how multiple different mechanisms, that might occur under specific genomic, phenotypic and microenvironmental contexts, ultimately result in the same search for proliferative and survival advantages (through activation of MYC), as well as immune escape and therapy evasion (from anti-CD20 biological therapies). Understanding the preferential strategies through which CLL cells hijack NOTCH1 signaling may present important clues for designing targeted treatment strategies for the management of CLL.

## 1. Introduction

Over the last three decades, many biological and genetic features of chronic lymphocytic leukemia (CLL) have been discovered to be of prognostic and predictive utility [1]. Among them, the most relevant and powerful are the somatic mutations of immunoglobulin heavy chain variable (IGHV) genes, chromosomal alterations (deletions of chromosomes 13q, 17p, and 11q, and trisomy 12), expression of the CD49d integrin and recurrent gene mutations of several genes including *TP53*, *ATM*, *SF3B1*, *BIRC3* and *NOTCH1* genes [2].

In particular, the interest around the Notch pathway in CLL [3,4,5] started to significantly grow after a 2009 pivotal study by Rosati and colleagues [6], who demonstrated that CLL cells are characterized by a constitutively activated Notch signaling. The study revealed a constitutively higher expression of the NOTCH1 and NOTCH2 receptors, as well as their ligands, compared to mature normal B lymphocytes. Furthermore, these NOTCH1-activated CLL cells showed increased survival and nuclear factor-kappa B (NF-kappaB) activity.

Soon after, the same group [7] and others [8,9] identified *NOTCH1* as one of the most frequently mutated genes at diagnosis, whose mutational burden can increase dramatically upon disease progression [10,11,12,13]. *NOTCH1* mutations displayed both prognostic and predictive role, as they both associate with poor outcomes [14,15] but also display a peculiar resistance to immunotherapy with anti-CD20 antibodies [16,17].

A few years later, a research by Fabbri and colleagues [18] reported the aberrant activation of NOTCH1 signaling in about 50% *NOTCH1*-wild type CLL from peripheral blood. This common activation of the NOTCH1 pathway, together with the frequent presence of somatic mutations, strongly hints at a significant biological role for this protein in CLL; however, despite a vast wealth of research, its functional role is not yet fully clarified and so for the oncogenic drives that favor the emergence of activation events. In addition, the peculiar dynamics that regulate Notch receptors and the mechanisms of signal transduction offer multiple layers of regulation, which are not completely elucidated in the context of CLL.

In this review, we will discuss some of the mechanisms of activation of NOTCH1 in CLL and the role of different players that can modulate the Notch pathway. First, we will present the details of structure and mechanisms of the NOTCH1 pathway, with particular attention to the multiple steps that are required for receptor activation and processing; then we will focus on CLL, to dissect the anatomy of *NOTCH1* mutations and their impact on the regulation and stability of NOTCH1 active protein and the main functional consequences on CLL pathobiology; finally, we will dive deeper in the other physiological and pathological mechanisms that drive Notch activation in CLL, beyond the presence of *NOTCH1* somatic mutations.

## 2. The Notch Pathway

The Notch pathway is a ubiquitous signaling system, highly conserved among metazoans [19], with a fundamental role in cell-fate determination in almost all developing tissues and organs [20,21,22] from embryonic development throughout adulthood [23]. In addition, it also functions to regulate tissue homeostasis and maintenance of stem cells in adults [22,24,25,26].

The network of Notch signaling is complex, and is the result of a highly coordinated work of many different molecules (receptors, ligands and interactors), which integrate multiple intracellular and extracellular signals and controls [27,28]. Thus the specific cellular environmental context, and different combinations of specific receptors/ligands interaction, may ultimately result in diverse outcomes (e.g., stem cell maintenance, progenitor selection, growth organizing boundaries, cell growth or inhibition, differentiation) [29,30].

In light of the ubiquitous physiological role of Notch signaling, genetic alterations of different pathway components can be found in a number solid tumors [31] and hematological malignancies [32], where Notch can act either as an oncogene or tumor suppressor [33]. In this perspective, the oncogenic role for Notch in lymphoid neoplasms is hinted by the fairly common presence of genetic aberrations in these tumors [34], as well as by the relevance of Notch signaling in the maturation and differentiation B and T lymphocytes [35,36,37]. Within the B-cell lineage, Notch signaling through NOTCH1 or NOTCH2 plays a critical role in the definition of different subtypes [38,39,40] possibly depending on the specific environmental context of different lymphoid niches [36]. In particular, NOTCH1 seems essential to steer differentiation of lymphoid progenitors toward a T-cell phenotype [35] whereas NOTCH2 seems required for marginal zone B differentiation [40,41]; however, the role of NOTCH1 is also evident in mature B cells and is significantly expressed in both naïve and memory B cells [18], which are the putative cells of origin of CLL [42].

From this perspective, the observations that Notch signaling in CLL resembles that of normal B cells favors the hypothesis of a repurposed physiological pathway to further promote survival and proliferation, rather than a *de novo* transcriptional program [18].

### 2.1. Structure of the Notch Receptors

In mammalian cells, the Notch system comprises four Notch paralogues, NOTCH1-4, which share most of their functional domains and present individual [43,44,45], yet sometimes overlapping [46], expression profiles and developmental functions. Different Notch genes have been implicated in various diseases and individual paralogs often have opposite effects even within the same system. In the lymphoid lineage, for example, NOTCH1 and NOTCH2 play radically different roles in the development of T and B lymphocytes [47,48,49]. Within the B-cell lineage, NOTCH1 and NOTCH2 are the most widely expressed receptors, although with different expression patterns throughout the maturation process from bone marrow to mature B cells; in contrast, NOTCH3 and NOTCH4 are barely expressed [50,51,52,53].

**Figure 1:**
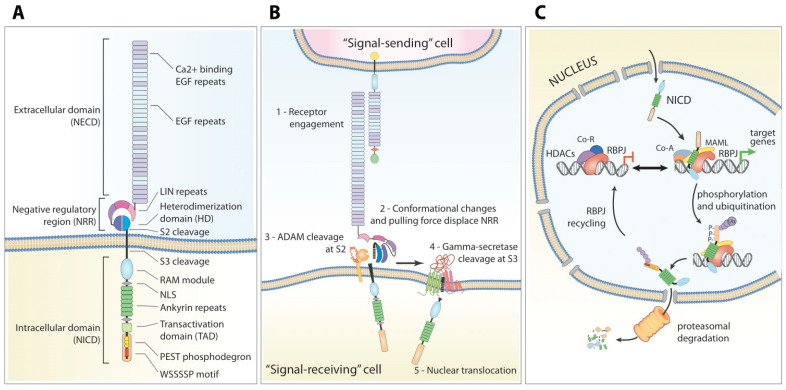
Structure and function of NOTCH1 signaling. (**A**) The NOTCH1 receptor consists of a single-pass transmembrane heterodimer organized in multiple functional domains, organized in three main regions: the extra-cellular domain (NECD), responsible for ligand binding; the negative regulatory region (NRR), which regulates receptor activation; the intra-cellular domain (NICD), which incorporates transcription effector functions. The NECD is essentially composed of 36 consecutive epidermal growth factor (EGF)-like domains responsible for ligand binding; the NRR is a beautifully simple but complex system of folded regions composed of three Lin12/Notch repeats (LNRs) that sterically protect the heterodimerization domain (HD). The NICD is composed by a protein-binding RBP-Jκ-associated molecule (RAM), seven ankyrin (ANK) repeats flanked by two nuclear localization signals, a transcriptional activation domain (TAD) and a glutamate, serine and threonine (PEST) domain. The PEST domain regulates NICD stability and degradation through multiple phosphosignals and the WSSSSP motif is recognized by E3 ubiquitin-ligases. (**B**) Mechanism of signal transduction. The ligand-engaged NECD is “pulled” toward the signal-sending cell (expressing the ligand) unfolding the three-dimensional protein structure of the NRR and exposing the HD. Nearby ADAM metalloproteases then cleave the NOTCH1 protein at the S2 site, whereas the gamma-secretase complex cleaves the S3 site. The liberated NICD is now free to translocate to the nucleus. (**C**) Nuclear functions and turnover of NICD. The translocated NICD binds with high affinity to the transcription factor RBP-Jκ; this displaces RBP-Jκ from other repressor proteins and histone deacetylases (HDACs), and favors binding of activating co-factors including Mastermind-like (MAML) and p300 which promote transcription. Within a few hours, the NICD is progressively phosphorylated and ubiquitinated, disengaged from RBP-Jκ, and destined for proteasomal degradation. RBP-Jκ is then bound by repressor proteins, shutting down the transcription program.

The basic structure of the NOTCH1 receptor consists in a single-pass transmembrane heterodimer, organized in multiple functional domains (Figure 1A). The NOTCH1 extra-cellular domain (NECD) is primarily responsible for ligand binding, and is composed of 36 consecutive epidermal growth factor (EGF)-like domains, some of which are calcium-binding and others are heavily glycosylated. The NECD is followed by three Lin12/Notch repeats (LNRs) and a heterodimerization domain (HD): these two modules act as a negative regulatory region (NRR), in which the LNRs sterically protect a buried cleavage site [54].

On the cytoplasmic side of the membrane, the Notch intra-cellular domain (NICD) is composed of a protein-binding RBP-Jκ-associated molecule (RAM), seven ankyrin (ANK) repeats flanked by two nuclear localization signals, a transcriptional activation domain (TAD), and a C-terminal region rich in proline, glutamate, serine and threonine (PEST). The PEST domain acts as a phosphodegron domain, as it contains multiple phosphosignals (reviewed in [55]) and motifs for substrate recognition by E3 ubiquitin-ligases that allow for fine-tuned regulation of protein stability and degradation [29,56,57].

Although very similar in structure, amino-acid composition and slight structural variations differentiate NOTCH2-3-4 from NOTCH1 (reviewed in [34] for B-cell malignancies), including the number of EGF repeats and the presence of TAD modules in the cytoplasmic domain, dramatically changing the function of each receptor.

### 2.2. Maturation and Processing

Notch receptors are first synthesized in the endoplasmic reticulum as a single, type-1 transmembrane precursor, and then transferred to the Golgi network for multiple post-translational modifications. Here, the NECD undergoes a complex maturation process, with the glycosylation of multiple EGF-like modules by the Fringe glycosyltransferases [58] Lunatic, Radical, and Manic Fringe, which can induce selective sensitivity to Jagged or Delta ligands [59]. Subsequently, the furin convertase operates the first proteolytic cleavage (named site-1 or S1) at the HD [60,61]: this process converts the single-chain protein into a heterodimer composed of the NECD and the transmembrane/intracellular (TMIC) C-terminal subunit. The two subunits are stabilized together at the HD through electrostatic interactions and non-covalent bonds with calcium ions [62]. The mature Notch heterodimer is then finally exposed on the surface of the cell outside lipid rafts [63].

Pulse-chase experiments demonstrated that there is a constant turnover of Notch receptors on the cell, with the disappearance of labeled Notch within hours [64]. If Notch does not engage a ligand, it is marked for degradation by E3-ligases such as AIP4/Itch [65] or Nedd4 [66] and internalized via early endosomal vesicles [67,68] with Numb-mediated AP2-clathrin adaptor-complexes [69].

### 2.3. Ligand-Induced Signaling Transduction

Notch signaling is initiated by the interaction of Notch receptors on a “signal-receiver” cell with transmembrane ligands exposed on adjacent “signal-sending” cells. The mammal Notch cognate ligands consist of five transmembrane proteins belonging to two distinct families: Jagged (JAG)-1 and-2 of the Serrate family and Delta-like (DLL) 1, 3 and 4 of the Delta family (reviewed in [70]).

Although these families share some structural homologies, they play fundamentally different roles as signals transmitted through Delta or Jagged can also differentially affect receiving cells within the hematopoietic system [71,72,73]; therefore, as the expression of different Notch receptors on lymphocytes depends on their maturation stage, different stimuli may result in different outcomes such as differentiation [74,75] or homeostasis [39].

Antiparallel engagement of a ligand by NOTCH1 is mediated by the NECD through its EGF repeats [76,77] and is characterized by different binding affinities for each ligand [78]. The physical interaction, coupled with the endocytosis of both Notch receptor and ligand by the respective cells [79], generates a mechanical force [80,81] that causes a conformational change of the NRR. This “stretching” unfolds the three-dimensional protein structure in the NRR [70,82], exposing a cryptic cleavage site (named site-2 or S2) on the HD [54]. Cleavage at the S2 site is performed by two non-redundant metalloproteases, ADAM10 and ADAM17 [83]. ADAM10 is required for ligand-dependent signaling, whereas ADAM17 seems responsible for ligand-independent NOTCH1 activation (Figure 1B) [84].

After proteolytic shedding of the NECD, the remaining TMIC fragment is readily cleaved on the cytoplasmic side at site-3 (or S3) by γ-secretase [85] at valine 1754 (coding reference UniProtKB-P46531). As a supramolecular protein complex, γ-secretase is mainly involved in the proteolysis of type I transmembrane proteins, composed of many different subunits, including presenilins (PSEN) 1 and 2, nicastrin, anterior pharynx defective 1 (APH-1), and presenilin enhancer 2 (PEN-2) [85] as well as plays a major role also within Alzheimer’s disease [86].

The S3 site represents an important site as the proteolytic cleavage generates a novel epitope, which can be recognized by several commercial antibodies. This allows for selective recognition of the NICD by Western blotting under denaturing conditions, which is the most widespread and reliable method [87,88,89,90]. Other studies have reported detection by immunohistochemistry, immunofluorescence and even flow cytometry [18,91,92,93,94]; however, these techniques require precise control of non-specific staining. Given the specificity of detection, the term “NICD” (along with its possible variations ICN, N-ICD, active ICN etc.) generally defines the functionally active cleaved form of NOTCH1, which will hereafter be used with this intended meaning.

Upon release within the cytoplasm, the NICD readily translocates to the nucleus [95,96] and interacts with DNA binding protein RBP-Jκ/CSL (CBF-1, Su (H), Lag-1) [97,98] via the RAM domain [86]. Further recruitment of other adapters such as MAML (Mastermind-like) induce conformational changes that release RBP-Jκ-bound co-repressors (SMRT, SKIP, SPEN, HDACs) [99,100], and replacement by co-activators (p300, PCAF and others) [101] to initiate target gene transcription (Figure 1C).

### 2.4. Ligand-Independent NOTCH1 Signaling

Beside this “canonical” Notch signaling, a wealth of evidence suggests that a number of mechanisms exist that activate Notch signaling independently of extracellular ligands (comprehensively reviewed in [102,103]). Although these mechanisms may be of limited magnitude compared to ligand-dependent signaling, they have proven to be of relevance. Many of these mechanisms revolve around the complex network of NOTCH1 intracellular trafficking (reviewed in [68]). Briefly, NOTCH1 receptors that undergo endocytosis and are exposed within lysosomes to acidic conditions [104] and lysozymes, may be proteolyzed at the NECD that extends into the intraluminal space, removing inhibitory sequences that prevent S2 recognition by γ-secretases [85,105,106]; further S3 cleavage would then release NICD into the cytoplasm [107,108,109].

Early studies [62,110] on the NOTCH1 receptor evidenced its high dependency on calcium ions to properly operate; consequently, Rand and colleagues demonstrated that calcium ions depletion induced the dissociation of Notch heterodimers [111]. In addition, varying intracellular pH and ion-concentrations (i.e., Ca^2+^, Zn^2+^) may also play a critical role in the stability of the receptor or affecting the activity of involved proteases [54,62,111,112].

**Figure 2:**
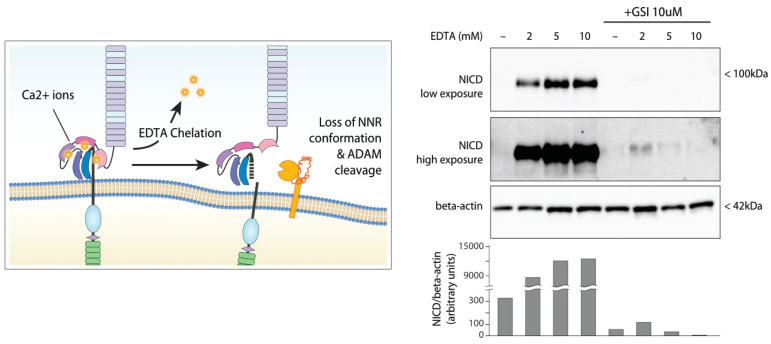
Ligand-independent NOTCH1 activation elicited by EDTA. Left panel: molecular interactions within the NRR are stabilized by divalent calcium ions; chelation by EDTA disrupts the three-dimensional conformation, allowing cleavage at S2 by ADAMs and subsequent gamma-secretase cleavage at S3 (not shown). Right panel: prototypic example of MEC1 cells treated with increasing concentrations of EDTA for 3 h showing evident accumulation of cleaved NICD, which is reversed by gamma-secretase inhibition (GSI). Original blots see Supplementary File S1.

In this regard, the most important stimulus for the NOTCH1 receptor is the chelating agent ethylenediaminetetraacetic acid (EDTA); EDTA is widely used as an anticoagulant in blood sampling procedures, as it also preserves blood cell morphology, and in cell biology to prevent clumping of cells grown in liquid suspensions, or detaching adherent cells for passaging. Millimolar concentrations of EDTA are sufficient to chelate the calcium ions that stabilize the NNR/HD interaction, causing its tertiary structure to unfold and exposethe S2 site [86] for ADAM17-dependent cleavage [84]; however, subsequent S3 cleavage still relies on the activity of gamma-secretases (Figure 2) [113] to promote NICD detachment and translocation.

One clear consequence is that CLL cells from blood samples collected in EDTA tubes are exposed to significant concentrations of chelating agent of about 6 mM, well above those employed for in-vitro stimulations [88]. Therefore, when investigating Notch signaling, proper control of the preanalytical phase becomes of paramount importance [114], as clearly reported by some studies [115].

## 3. NOTCH1 Mutations in CLL

### 3.1. General Features

In human cancers, somatic mutations of *NOTCH1* generally cluster in two main hotspots: the HD and PEST domains. This distribution reflects the functional importance of the affected domains that regulate, respectively, activation and stability. HD mutations are mostly common in T-ALL, where they often represent the primary oncogenic lesion and can be found in up to 60% cases. These are mostly missense mutations that alter the three-dimensional conformation of the NRR, allowing for ligand-independent S2 and –S3 cleavage, resulting in constitutive activation of the Notch pathway.

In contrast, CLL is strongly characterized by the other class of *NOTCH1* mutations, which are restricted to the TAD and PEST domains; therefore, NOTCH1 signaling in CLL still requires external receptor engagement for cleavage to be triggered. Because of this mechanism of action, *NOTCH1* mutations in CLL are mostly passenger events, acquired during the course of the disease and are rarely found within precursor populations [116,117,118].

The reason for such a striking difference in mutational hotspot usage is still unknown. However, given the different roles of NOTCH1 signaling in T- and B-cell maturation, specific microenvironmental conditions and layers of epigenetic regulation may induce selective pressure to push cells to select the most advantageous alteration: a ligand-independent, constitutive pathway activation for Notch1-addicted T cells versus a ligand/microenvironment-dependent activation for B cells.

### 3.2. Coding Mutations

**Figure 3:**
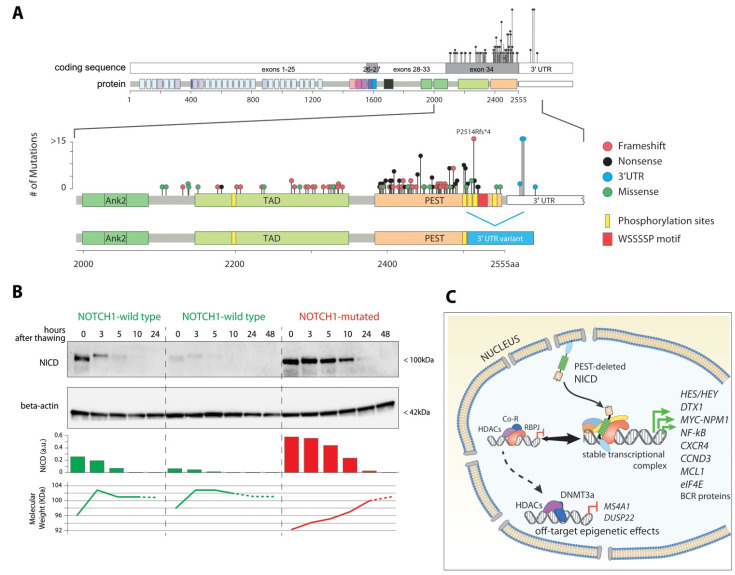
NOTCH1 mutations in CLL. (**A**) In CLL, *NOTCH1* mutations almost exclusively affect the c-terminal domain of the protein, specifically exon 34. The most common mutation is c.7514–7542delCT, p.P2514Rfs*4, which truncates the phosphorylation and ubiquitination (WSSSSP) sites. Other mutations within the coding sequence are mostly frameshift (red) and nonsense (black) truncating events scattered across exon 34, leading to the same outcome. The 3′UTR mutations (blue) lead to an alternate splicing event, mostly with a cryptic site around Q2503, which leads to an alternate transcript again lacking the main phosphodegron. Missense mutations (green) are rare events, usually private polymorphisms. (**B**) Prototypic immunoblot detection of NICD in primary CLL samples. Wild-type NICD is promptly phosphorylated and ubiquitinated, with an increase in molecular weight, degradedation within hours. Mutated NICD (p.2514R*fs4) displays prolonged stability. Dashed lines represent a hypothetical trajectory since protein levels were undetectable. Samples were thawed cryopreserved PBMC from EDTA-stabilized peripheral blood samples. Densitometry and molecular weight calculations were performed with ImageLab (Bio-Rad). (**C**) Schematic representation of the effect of *NOTCH1* mutations. Superstable NICD skews the balance of the RBP-Jκ complex toward transcriptional activation, enhancing gene transcription of multiple genes. Unbound repression proteins, mostly epigenetic modifiers such as histone deacetylases (HDACs) and methyltransferases (DNMT3a) relocate to other targets. Original blots see Supplementary File S1.

From a genomic point of view, all *NOTCH1* mutations in CLL occur within exon 34, which encodes for about half of the NICD, specifically the TAD and the PEST functional domains, responsible for Notch transcriptional activity and stability (Figure 3A).

The most common is a 2-bp deletion occurring at position c.7541–7542 (NM_017617.5), a variant commonly known as “delCT”; this variant, first identified in 2009 by Di Ianni and colleagues [7], results in a premature stop codon after proline 2514 (p.P2514Rfs*14), which is the primary phosphorylation site required for NICD ubiquitination (Figure 3A).

The advent of next generation sequencing (NGS) rapidly allowed a more thorough examination of the mutational profile of the *NOTCH1* gene: besides confirming the recurring presence of the delCT mutation, these studies identified other less frequent variants within exon 34 [8,9,119]. These variants, instead of being constrained to specific hotspots like the delCT, are found scattered across the whole of exon 34, and are highly enriched in nonsense and frameshift events. From a functional perspective, these mutations ultimately result in the translation of a truncated protein, with the loss of the phosphodegron sites around serines 2513/2517 and the WSSSSP motif at position 2520–2525. These domains are required for targeting the NICD for ubiquitination and subsequent proteasomal degradation, and thus the mutated protein fails to be ubiquitinated, displays abnormal stability and accumulates within the cell, prolonging the activation of NOTCH1 signaling (Figure 3B) [120].

Overall, the incidence of these mutations varies at different disease stages, ranging from about 3–11% at the MBL stage [121,122], to 15–25% at CLL diagnosis [10,11], and 20–40% for refractory/relapsed CLL and Richter syndrome [12,13]. In addition, the average mutational burden can vary greatly, has evolved over time in parallel with technological advancements such as NGS [10,11] and droplet digital PCR [123,124,125]. In particular, the latter studies report a detection limit of 0.03% for variant allele frequency, way lower than the NGS limit of about 0.3–1.3 [11,126]; in parallel, they have also suggested that *NOTCH1* delCT mutations may be present in a greater fraction of CLL cells, ranging from 18.6–25% [124,125] up to 55% in CLL cases with trisomy 12.

At the other end of the spectrum, missense mutations are a much rarer event and, rather than acquired somatic variants, they often represent private germinal polymorphisms [11]. In contrast to truncating mutations, missense mutations change only the affected amino acid, leaving the rest of the protein fully intact. At present, the functional impact of these mutations is unknown, since they usually do not seem to disrupt functional motifs and do not cluster at specific positions [11] suggesting a limited, if negligible, role in the course of CLL.

### 3.3. Non-Coding Mutations

Beside the known mutations within the coding region, a new and unexpected type of alteration was identified by whole-genome and whole-exome sequencing. In 2015, Puente and colleagues [127] identified in their datasets several unusual splicing isoforms of *NOTCH1*, carrying somatic mutations in the 3′ untranslated region (UTR) corresponding to positions 7668 + 371A > G, 7668 + 378A > G and 7668 + 380A > C. These point mutations generate a novel motif recognized as a splicing acceptor which, in turn, either interacts with exon 33 (splicing out the whole of exon 34) or, more frequently, triggers a cryptic splicing donor within exon 34 around glutamine 2503 (Figure 3A). Once again, the resulting protein loses the functional PEST domain, displays prolonged stability, and accumulates within the cell [128,129]

The impact of these mutations was later confirmed in further studies and larger cohorts with a frequency of about 2–4% and a prognostic impact similar to *NOTCH1*-delCT-mutated CLL, for both time-to-first-treatment and overall survival [10,11,130].

## 4. Functional Consequences of NOTCH1 Mutation in CLL

At difference with the fairly straightforward landscape of mutations, the phenotypic consequences of the accumulation of an active NICD in CLL cells have been, for a long time, far from clear. Many groups have investigated the different lights and shadows of Notch signaling in CLL cells, without identifying a single, major, unified mechanism of action. Rather, the resulting picture highlights a number of different mechanisms (extensively reviewed in [131]) that might occur under specific genomic, phenotypic and microenvironmental contexts [87,88,132,133,134,135,136].

### 4.1. Proliferation and Metabolism: A Story of MYC

Over time, one of the most recurrent and reproducible phenotypic effects of prolonged NOTCH1 signaling in CLL was the capability to induce the well-known oncogene MYC. MYC is a basic helix-loop-helix leucine zipper transcription factor that regulates a large number of diverse target genes involved in proliferation and metabolism, and due to its role as a master regulator of cell growth, is regarded as one of the most important oncogenes [137].

Not long after the *NOTCH1* lesions were identified in T-ALL [138], NOTCH1 signaling was shown to upregulate MYC expression, by direct interaction with several regulatory regions in proximity to the *MYC* promoter [139,140,141]. Further studies demonstrated the existence of NOTCH1-controlled enhancers downstream of the *MYC* locus, that were restricted to T-ALL and responsible for the NOTCH1-dependent activation of *MYC* [142,143,144]. Driven by these observations, we also investigated whether aberrant NOTCH1 signaling could impact on cell growth and proliferation, demonstrating in the context of CLL that NOTCH1 can directly bind regulatory elements at the *MYC* locus and induce gene transcription [88]. By gene expression profiling, we also showed that this NOTCH1/MYC activation upregulates genes related to ribosome biogenesis such as nucleophosmin 1 (NPM1) and ribosomal proteins (RNPs), potentially conferring cell growth and/or proliferation advantages on *NOTCH1*-mutated CLL cells. Our study also confirmed a previous observation by Jitschin and colleagues [133], who demonstrated that direct interaction of CLL cells with stromal cells induced NOTCH1-related *MYC* expression and a metabolic shift from mitochondrial respiration to glycolysis (Warburg effect), which can provide growth and survival advantages for tumor cells [145].

The capacity of NOTCH1 to induce *MYC* expression was also confirmed by later studies in CLL [57,145,146], mantle cell lymphoma (MCL) [147] and other non-hematological cancers [148,149,150,151]. In particular, the work of Ryan et al. [147,152] revealed that the NOTCH1-responsive element active in MCL (and possibly also in CLL) is different from the one identified in T-ALL, located about 500 kb upstream of the MYC locus, and its usage is restricted to B cells. The same mechanism was reported also for CLL by Fabbri and colleagues [18]. Using ChIP-seq experiments, they confirmed not only the capacity of NOTCH1 to induce *MYC* transcription, but the usage by NOTCH1 of the same B-cell specific 5′ enhancers previously described in lymphoma; interestingly, copy-number analyses suggested that this enhancer region is recurrently affected by focal duplications, mutually exclusive with *NOTCH1* mutations.

### 4.2. The Complex Interplay with NF-kappaB

Activation of NOTCH1 signaling is usually correlated with the activation of the NF-kappaB pathway, which is also critically involved in CLL pathogenesis [153,154,155].

Notch/NF-kappaB interplay has been documented in different malignancies, such as T-ALL [156] and Hodgkin Lymphoma [157], but also in the physiological maturation of B cells [158], where NF-kappaB can trigger the Notch signaling pathway in neighboring cells to induce expression of JAGGED1 protein [159].

The possibility that NOTCH1 activates NF-kappaB in CLL was suggested by early evidence [6] which reported a higher degree of NF-kappaB-binding to its consensus sequence by electrophoretic mobility shift assay; further studies seemed to confirm these observations, evidencing the activation of the NF-kappaB pathway especially in the context of *NOTCH1*-mutated CLL cases, in which the activity of NICD was enhanced due to its prolonged half-life [160].

Although the mechanisms of NOTCH1/NF-kappaB interaction in CLL are rather elusive [161], evidence in models of T-cell leukemia suggest the possibility of both a transcription-dependent interaction, through its target *HES1* that, in turn, can modulate other components of NF-kappaB pathway [156], and a transcription-independent interaction, where NOTCH1 may directly interact with the p50/c-Rel subunit, to retain the active NF-kappaB heterodimer in the nucleus [162].

We observed nuclear retention of NF-kappaB subunits in the context of CLL [136], by taking advantage of a NICD-transfected CLL cell line model. In this study, we could demonstrate that overexpression of a mutated, stabilized form of NICD resulted in nuclear retention of the RelA/p65 subunit, triggering the NF-kappaB canonical pathway. In turn, this activation was associated with elevated levels of integrin CD49d, an established key regulator of microenvironmental interactions and a negative prognosticator in CLL [163,164].

### 4.3. Nuclear Rewiring Triggers Epigenetic Downregulation of CD20: Clinical Implications for Anti-CD20 Immunotherapy

Soon after *NOTCH1* mutations were identified in CLL, several retrospective analyses demonstrated that *NOTCH1*-mutated patients have inferior survival and worse treatment outcomes compared with *NOTCH1* wild-type patients [11,14,15,165,166,167,168,169,170], albeit with discordant results regarding the impact on overall survival or progression-free survival [16,170,171,172].

An unexpected observation came from the CLL8 clinical trial, which compared fludarabine/cyclophosphamide versus fludarabine/cyclophosphamide plus rituximab. Surprisingly, patients carrying *NOTCH1* mutations seemed not to benefit from the addition of rituximab to the backbone therapy, displaying a progression-free survival significantly lower than *NOTCH1*-wild-type cases and comparable to those treated without rituximab [16]. These data were subsequently confirmed in a homogeneously prospective CLL series that underwent rituximab consolidation after first-line therapy; in this study, patients carrying *NOTCH1* mutations were characterized by lower rate of complete remission and response duration while in rituximab maintenance [17]. Similar results came from trials with the second generation anti-CD20 monoclonal antibody, ofatumumab [173], whereas no major differences were found with the third-generation antibody, obinutuzumab [174].

These clinical observations led us to investigate the putative underlying mechanisms that could explain such behavior. We demonstrated that *NOTCH1*-mutated CLL cells are characterized by lower CD20 expression [128,134,175] and lower sensitivity to rituximab-mediated complement-mediated cytotoxicity [134]. This effect was particularly evident in CLL cases that also carried trisomy 12, which are characterized by a peculiar phenotype and elevated CD20 expression [176,177]. In our study, we showed that accumulation of mutated NICD in the nucleus is responsible for a dysregulation of histone deacetylases (HDACs)-mediated epigenetic repression of *MS4A1*/CD20 transcription. Specifically, in a condition of NICD accumulation (via transfection with an exogenous expression vector), RBP-Jκ showed preferential binding to NICD, rather than HDACs, in an abnormally stable transcription complex (Figure 1C). This has an impact on the amount of free HDACs that can bind to and epigenetically silence other genomic regions [134], since chromatin immunoprecipitation experiments demonstrated that, in those NICD-accumulating cells, the *MS4A1* promoter displayed a higher degree of bound HDACs compared to untransfected cells.

The rewiring of nuclear epigenetic circuitries influencing CD20 expression was later found to influence other targets, such as DNMT3A, to ultimately upregulate *CCR7* expression and increase CCL19-driven homing to lymphoid niches. These results suggest that *NOTCH1*-mutated CLL cells may be facilitated at homing to privileged niches that provide pro-survival stimuli, further fueling pathway activation [135].

## 5. Other Mechanisms of NOTCH1 Activation in CLL

### 5.1. Evidence of NOTCH1 Sustained Activation in the Absence of Genetic Mutations

The first consistent report on the activity of the Notch pathway in CLL [6] revealed that expression of NOTCH1, NOTCH2 and their ligands Jagged 1/2 was common and at odds with normal B lymphocytes, possibly due to deregulated protein turnover rather than differences in gene transcription and/or mRNA stability.

Although the majority of functional studies have been performed on peripheral blood, given the ease of access and sampling, several studies managed to investigate NOTCH1 signaling within the lymph nodes. Consistent with microenvironment-driven activation of NOTCH1, these investigations reported a significant expression of activated NOTCH1 within the lymph node microenvironment compared to peripheral blood [87,91,178] and co-cultures with stromal cells [53,87,133]. In addition, Fabbri and colleagues [18] demonstrated that Notch signaling was particularly active in the mantle zone but not within germinal center-cells. These data provide evidence that NOTCH1 signaling is indeed activated in naïve and memory B cells, which are the putative compartments-of-origin of CLL.

Surprisingly, the same paper [18] reported that about 50% *NOTCH1*-wild type CLL from peripheral blood presented detectable NICD staining in immunoblots and immunofluorescence. Since the known half-life of the NICD-RBP-Jκ-CoA activation complex is estimated in a few hours [179,180] after a single round of stimulation (Figure 3B), these data suggest that some kind of prolonged receptor engagement must be in action. Interestingly, the transcriptional signature of these *NOTCH1*-wt/ICN + CLL samples was consistent with that of *NOTCH1*-mutated samples and similar to that of normal B-cell counterparts upon stimulation.

The co-expression of Notch and Jagged proteins in CLL cells [6] suggest a role for cis-interaction, i.e., autocrine interaction of ligand-receptor on the same cell. However the current consensus model for Notch activation requires an interaction between two cells, i.e., in trans, for receptor activation, whereas a ligand-receptor interaction on the same cell, i.e., in cis, mostly results in an inhibitory effect [181,182,183,184]. It has recently been shown that a cis-interaction may be activating under certain conditions, but these data need further validations [185]. In the context of CLL, cis-interactions would be an attractive hypothesis, but the diverse signaling intensities reported in peripheral blood versus lymph node seem to argue against this scenario [87,178].

**Figure 4:**
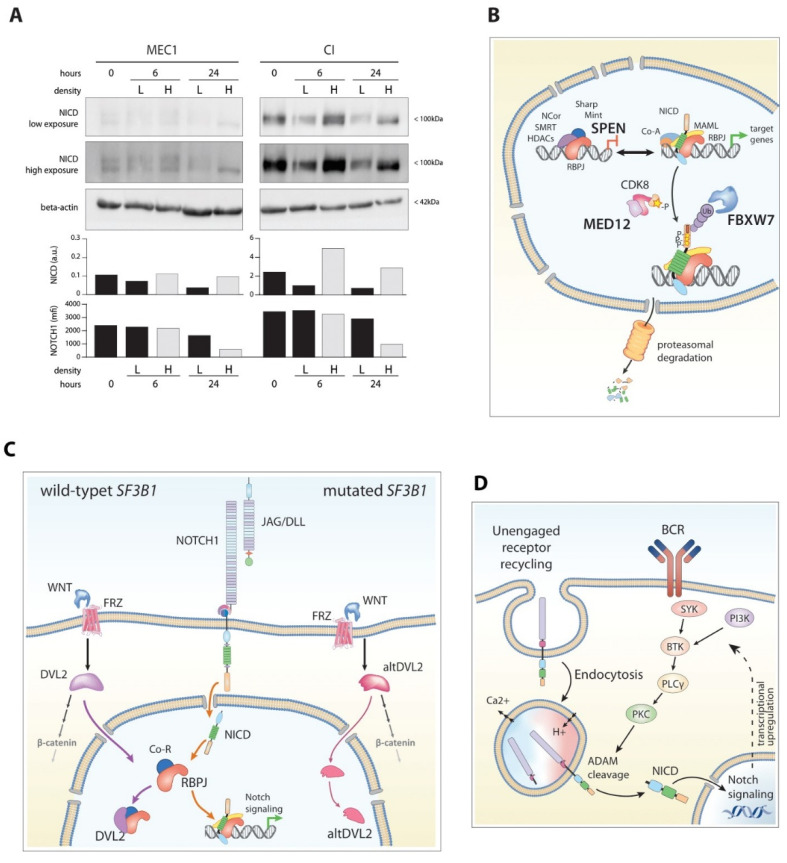
Other mutation-independent mechanisms of NOTCH1 in CLL. (**A**) NOTCH1 activation is mostly dependent upon microenvironmental interactions. Immunoblot and flow cytometric detection of NICD in *NOTCH1*-wild-type (MEC1) and *NOTCH1*-mutated (3′UTR mutation, CI) CLL-like cell lines, cultured at low (L, 0.054 × 10^6^ cells/cm^2^) and high (H, 3 × 10^6^ cells/cm^2^) concentration for the indicated time. Samples at time zero were collected after an overnight culture at low concentration. For the high exposure blot, a gamma correction of 0.5 was applied to enhance visibility. Blots are derived from one single gel and are separated only for presentation purposes. (**B**) Other modulators of NOTCH1 signaling recurrently mutated in CLL. FBXW7, MED12 and SPEN are highlighted in bold. (**C**) Putative model for the interaction between Wnt and Notch pathways mediated by DVL2 in the context of *SF3B1* mutations. DVL2, a key component of the Wnt pathway, is reported to act as a negative regulator of NOTCH1 either by direct interaction with NICD (dashed lilac line) or by sequestering RBP-Jκ, reducing its bioavailability to NICD (solid lilac line). SF3B1 mutations induce alternative splicing of DVL2 (altDVL2) which is less proficient in the interaction with RBP-Jκ (solid pink line, right side). (**D**) Proposed model of BCR-mediated NOTCH1 activation. Unengaged Notch receptors are internalized for recycling; chemical adjustments in the endocytic compartment, with progressive acidification and efflux of calcium result in destabilization of the NRR and prime the receptor for cleavage. Active PKC, elicited by BCR signaling, triggers co-endocyted ADAM-metalloproteases which initiate processing of the Notch receptor. Abbreviations: a.u, densitometric arbitrary units; mfi, mean fluorescence intensity; SF3B1, splicing factor 3 subunit 1; WNT, wnt ligands; FRZ, Frizzled receptors; DVL2, dishevelled 2; altDVL2, alternate splicing of DVL2;. BCR, B-cell receptor; SYK, spleen associated tyrosine kinase; BTK, Bruton’s tyrosine kinase; PI3K, phosphoinositide 3 kinase; PKC, protein kinase C; PLCγ, phospholipase C γ. Original blots see Supplementary File S1.

Indeed, MEC1 cells cultured in-vitro at high concentrations (~12 × 10^6^/mL, 3 × 10^6^/cm^2^) to favor cell-cell interactions, show significant cleavage of NOTCH1 as well NICD induction, whereas sparse cells (~0.375 × 10^6^/mL, 0.054 × 10^6^/cm^2^) maintained almost constant levels of NOTCH1 and shut down NICD expression (Figure 4A). Of note, incubation with gamma-secretase inhibitors (DAPT) was able to prevent cleavage of NICD in dense cultures but had no significant effect on sparse cultures.

Nevertheless, the complexity of Notch signaling and the intertwined signaling circuitries provide a number of other mechanisms, both activating and suppressing, that can result in the activation of the NOTCH1 pathway, which will be discussed below.

### 5.2. Recurrent Mutations of Modulators of NOTCH1 Signaling

#### 5.2.1. FBXW7

One of the key regulators of NOTCH1 activity is the E3 ubiquitin-ligase FBXW7 (F-box with seven tandem WD40). F-box proteins are responsible for regulating the turnover of their targets: they recognize their substrates through the presence of conserved phosphodegron motifs that are phosphorylated at specific residues; the substrate is then ubiquitinated and targeted for proteasome degradation [186].

FBXW7, in particular, regulates NOTCH protein activity by controlling its half-life, maintaining optimum protein levels in the tissue. It is perhaps the most widely studied F-box protein due to its role in a panel of both normal and malignant cellular processes [187,188]; in fact, mutations of the *FBXW7* gene have been identified in more than 30% of pediatric T-ALL [188,189] and several other hematological and non-hematological diseases [190,191,192,193,194] These mutations disrupt the direct interaction between FBXW7 and NICD and extend the half-life of NICD, thus mimicking the effects of canonical *NOTCH1* mutations (Figure 4B). For example, T-ALL cells harboring *FBXW7* mutations are generally resistant to GSI [180,188].

In CLL, mutations of *FBXW7* have been identified with a frequency estimated by early studies of about 2–5% cases [118,169,195,196,197] that was later confirmed by more recent studies [170,198]. Like *NOTCH1* mutations, *FBXW7* mutations also show a distinct association with trisomy 12 CLL [169,170] and the two are thought to contribute to the transformation of CLL to Richter syndrome [195]. Nevertheless, these mutations are mostly mutually exclusive, suggesting a functional redundancy, since both mutations lead to NICD stabilization with an equivalent oncogenic effect over time.

A recent study by Close and colleagues [57] investigated in detail the role of *FBXW7* mutations in CLL. The most frequently affected residues correspond to the three arginine residues (465/479/505) required for substrate recognition, which are collectively mutated in about 50% of all *FBXW7*-mutated cases. These substitutions, which very likely alter the electrostatic/hydrophobic interactions between FBXW7 and its substrates, could prevent NICD degradation, even after inhibition of translation.

However, to make the landscape even more complex, FBXW7 is known to regulate the activity of a much wider range of substrates through the ubiquitin-proteasome system-mediated degradation pathway and. Targeted knock-outs have suggested that there may be close to 90 FBXW7 substrates [199], including MYC, cyclin E, mTOR, c-Jun, MCL1, hypoxia-inducible factor 1-α (HIF1-α), and AURKA. In particular, the MYC protein, which has a characteristic very short half-life (as is the case with NICD), is a recognized target of FBXW7 [200], and T-ALLs with *FBXW7* mutations not only show prolonged NOTCH1 signaling, but also MYC stabilization and increased protein levels [180]. In contrast, the off-NOTCH1 effects of *FBXW7* mutations in CLL seem more elusive. Despite in vitro luciferase reporter assays demonstrating elevated MYC promoter activity, transcript and protein levels were not dissimilar between *FBXW7*-mutated and -unmutated cases [57], suggesting additional layers of regulation. Furthermore, *FBXW7* mutations were associated with elevated levels of HIF1-alpha, but not Cyclin E or NF-kappaB2/p100. Although all of these proteins have been shown to be of relevance in CLL [57,201], these data suggest a rather restricted impact of these mutations on the protein interactome, at least within CLL.

Beside FBXW7, other regulators of NOTCH1 signaling have been found mutated in CLL at various frequencies, specifically MED12 and SPEN.

#### 5.2.2. MED-12

Mediator complex subunit 12 (MED12), together with MED13, CyclinC and CDK8, is part of the kinase module of the Mediator complex, a central integrator and processor of polymerase II transcription [202]. This complex transduces information conveyed by transcription factors to promote long-range chromatin interactions (via looping) and the formation of transcription pre-initiation complexes [203].

The MED12-Cyclin C-CDK8 complex is also responsible for the turnover of the NICD, since it is directly involved in phosphorylating the PEST domain, which acts as the targeted substrate of FBXW7 (Figure 4B) [204,205]. Mutations in *MED12* have been identified by several studies in 1–9% of CLL cases [127,196,206,207,208,209,210]. *MED12* mutations, like *FBXW7* mutations, were mutually exclusive of *NOTCH1* mutations but still associated with trisomy 12 [89]. Biochemical studies revealed that *MED12* mutations lead to decreased CDK8 kinase activity by disrupting the MED12-Cyclin C binding interface [207] and causing abnormal accumulation of NICD [89].

#### 5.2.3. SPEN

The *SPEN* gene encodes for an adaptor protein part of the Mint/SHARP/SPEN complex. This system interacts with several transcription factors, including Msx2 and RBP-Jκ, and acts as a bridge between RBP-Jκ and repressor proteins, such as NCor/SMRT/HDACs (Figure 4B) [211,212]. Prolonged absence of NICD is believed to allow recruitment of the adaptor to RBP-Jκ, and then of the repression complex, to condense the chromatin around NOTCH1 target genes and inhibit gene expression.

Inactivating mutations of *SPEN* have been identified in CLL at a low frequency between 1–10% [115,119,127,198,213], and functional studies have confirmed an association with increased NOTCH1 activation. In particular, Edelmann and colleagues [115] highlighted that *SPEN*-mutated tumors had significantly higher median expression levels of NOTCH1 target genes, as well as frequently higher expression of MYC.

Although the overall mutation frequency of these NOTCH1-related genes is quite low, when taken together, they may account for an additional 5–10% of CLL cases with a possible NOTCH1 dysfunction. Helbig and colleagues [198] reported that the combination of these NOTCH1 regulatory pathway mutations present a clinical impact not dissimilar to *NOTCH1* canonical mutations, although small numbers do not easily allow for an estimation of their independent prognostic impact. Nevertheless, these recent reports highlight the importance of performing molecular investigations in a pathway-wise manner instead of focusing on single genes to possibly avoid underestimating the biological impact of multiple lesions converging on the same oncogenic pathways.

### 5.3. Mutations of SF3B1: An Interplay with the Wnt Pathway

The landscape of genetic lesions involved in dysregulating NOTCH1 signaling was unexpectedly enriched by mutations in the RNA splicing factor 3b subunit 1 (SF3B1). SF3B1 is a key component of the splicing machinery, responsible for recognizing the branchpoint sequences in proximity of the 3′ splice site (acceptor site) and allowing intron removal from precursor-messenger RNAs.

Mutations of *SF3B1*, found in about 10% CLL cases, are predicted to alter the protein’s tertiary structure, hampering the correct high-affinity recognition of the substrates and resulting in the selection of alternative 3′ splice sites. In CLL, mutations of *SF3B1* have been shown to induce transcriptome-wide alterations, with an increased frequency of alternative 3′ splice site selection and functional consequences for several pathways, such as DNA damage, telomere maintenance, and methylation [10,214,215,216,217].

The pivotal study by Wang and colleagues [216] on the transcriptome-wide alterations induced by *SF3B1* mutations identified consistent splicing alterations of the dishevelled-2 (*DVL2*) gene. DVL2 is a key mediator of the Wnt pathway and has demonstrated the ability to act as a negative regulator of the NOTCH1 pathway by binding to RBP-JΚ and/or NICD itself. By exploiting both a Notch luciferase-reporter-assay system [218] and transfection of synthetic constructs, they detected significantly higher Notch pathway activation; furthermore, overexpression of wild-type DVL2 repressed NOTCH1 signaling [219] and expression of altered DVL2 counteracted this effect, suggesting a dominant impact of the alternatively-spliced isoform (Figure 4C).

In our subsequent study [175], we moved to primary CLL cells and used gene expression profiling to show that *SF3B1*-mutated cases share a gene signature with *NOTCH1*-mutated cases that drove an unsupervised co-clustering of the two categories with respect to wild-type cases. Moreover, increased NOTCH1 signaling positively correlated with the expression of altered DVL2 and reduced CD20 expression in *SF3B1*-mutated CLL cases, suggesting that *SF3B1* mutations represent another factor associated with the reduction of CD20 expression through mutation-independent activation of the NOTCH1 pathway.

The association between mutated *SF3B1* and activation of the NOTCH1 pathway has also been investigated at the epigenetic level. Pacholewska and colleagues investigated DNA methylation profiles in *SF3B1*-mutated CLL patients, identifying differentially methylated regions associated with multiple cancer-related signaling genes, including NOTCH1, and enriched within the NOTCH signaling pathway [217].

### 5.4. DNMT3a

DNA methyltransferase 3A (DNMT3A), along with DNMT3B, is responsible for establishing the patterns of DNA methylation early in embryogenesis through de novo methylation of unmethylated CpG sites, wheras DNMT1 maintains these patterns throughout cell division [220].

Although *DNMT3A* mutations are frequent in acute myeloid leukemia, myeloproliferative disorders, and T-ALL [221], it is not an expected lesion within CLL; instead, several works demonstrated that low activity and expression of DNMT3A is important for CLL pathogenesis and evolution [222,223,224], and mouse models with deletion of Dnmt3a consistently developed CLL [225].

A very recent study [226] highlighted a possible role for DNMT3A in modulating the NOTCH1 pathway. Expression of DNMT3A is highly variable in CLL cells; nevertheless, lower DNMT3A expression is associated with a more aggressive disease, presenting a shorter failure-free survival. Therefore, Biran and colleagues generated a mouse model with B-cell restricted knockout of Dnmt3A. This model showed very high penetrance of CLL, and the disease was characterized by focal hypomethylation and activation of Notch and MYC signaling pathways, allegedly via the direct hypomethylation of gene promoters. Furthermore, MYC amplification on chromosome 15 was detected in all sick mice, and their spleens were characteristically infiltrated by high-MYC-expressing CLL cells. Interestingly, Dnmt3a-depleted CLL was sensitive to pharmacologic inhibition of Notch signaling in vitro and in vivo, supporting hyperactivation of Notch signaling in these cells and a high dependency on this pathway for survival. These insights provide a novel mechanism linking epigenetic alterations in CLL to Notch signaling and a potential animal model to investigate non-mutational Notch activation.

### 5.5. Bidirectional Interplay between NOTCH1 and BCR Pathways

One of the fundamental signaling axes in CLL cells is the B-cell receptor, which mediates extrinsic and autonomous signals that promote cell survival and proliferation [227,228]. Early evidence suggested that NOTCH1 signaling could influence the expression of multiple genes encoding elements of the BCR pathway, such as LYN, SYK, BLNK, and PIK3γδ [18,147] and, in turn, appeared as an important mediator of BCR-induced B-cell activation in murine primary B-cells [229].

Del Papa and colleagues [230] reported evidence of a relationship between the BCR pathway and NOTCH1 activity, since BCR stimulation was able to increase NICD expression that was counteracted by ibrutinib treatment. Furthermore, ibrutinib-treated CLL patients showed progressive downregulation of NOTCH1 activity during therapy, which was restored at relapse and remained activated in ibrutinib-resistant disease.

A subsequent study by Arruga and colleagues [90] further demonstrated that this relationship involves functional cooperation of the NOTCH1 and BCR pathways connected by a feed-forward loop. In this study, BCR cross-linking by anti-IgM antibodies resulted in an increase in surface NOTCH1 levels, but not at the transcript level, suggesting a mechanism of post-transcriptional regulation. Furthermore, upregulation of NOTCH1 expression was paired with upregulation of the target genes *DTX1* and *HES1*, and these effects were reversed by ibrutinib and gamma-secretase inhibitors.

A possible key molecule involved in this circuit has been identified in protein-kinase C (PKC). Un-engaged Notch receptors on the cell surface are internalized for endosomal degradation. The endocytic vesicles progressively experience a low-pH and low-calcium environment, which may facilitate denaturation of the NECD within, exposing the S2 and S3 sites to ADAM10/17-mediated cleavage [231]. However, activation of ADAM10/17 seems to be influenced by the presence of active and mature PKC [231], since pharmacological treatments with its agonists (PMA) or antagonists (sotrastaurin) were able to modulate Notch processing [90,231] (Figure 4D).

These data were somewhat confirmed in the context of CLL [90], where PMA could efficiently trigger ligand-independent cleavage of NOTCH1 and promote *HES1* and *DTX1* transcription. This observation allows us to hypothesize a strong functional connection between the two pathways: although the former can induce ligand-independent NOTCH1 activation, the latter has direct effects on IgM signaling, further enhancing anti-IgM responses [90]. It has to be noted that these results were mostly significant within *NOTCH1*-mutated CLL, which are more prone to display significant and prolonged changes, whereas wild-type cases were less responsive, possibly due to the rapid degradation of NICD.

The presence and relevance of a functional connection between the BCR and NOTCH1 pathways in CLL is also supported by the lower redistribution of lymphocytosis and lower nodal shrinkage of *NOTCH1*-mutated CLL during ibrutinib treatment [232], and the peculiar association of *NOTCH1* mutations with a specific stereotyped configuration of the BCR (IGHV4-39/IGKV1 (D)-39) defined as subset 8, which is characterized by robust BCR signaling and exhibits the highest risk for Richter transformation [233].

### 5.6. AKT towards Richter Syndrome

One last, recently published, piece of evidence has uncovered a functional connection between Notch signaling and the AKT pathway. Although novel in the CLL setting, such AKT-NOTCH1 interactions have been previously reported in drosophila and T-ALL [234,235].

In their study, Kohlhaas and colleagues [236] demonstrated that B cell-restricted activation of AKT in the eµ-*TCL1* mouse model drives transformation to an aggressive lymphoma. This evolution closely resembled Richter transformation (RT), with an intermediate phenotype between CLL and DLBCL, massive splenomegaly, and reduced overall survival. The authors observed that transformed B cells with active AKT signaling showed upregulation of genes associated with Notch signaling; specifically, they detected increased expression of NOTCH1 and NOTCH3 receptors, as well as increased intracellular NICD in adult mice with a fully transformed RT phenotype. Interestingly, they also observed increased levels of Notch Dll1 ligand on CD4^+^ FoxP3^+^ T cells, but not on other cell types, and suggest that constitutive Akt activation may induce expansion of T cells overexpressing Dll1, in turn sustaining Notch1 activation and facilitating CLL to RT transformation.

These results, in line with the established role of NOTCH1 signaling in RT [237,238], suggest that constitutive AKT activation may initially amplify NOTCH1 signaling or add additional signals that accelerate transformation. These signals may later further cooperate with upcoming *NOTCH1* mutations at later disease stages to increase survival and immune escape [239,240].

## 6. Conclusions

Deregulation of NOTCH1 signaling is one of the most consistent results in the last decade of CLL research. In particular, the proof of aberrant activation was first presented by Rosati and colleagues, even before the discovery of *NOTCH1* mutations [6]. The notion of a significantly deregulated NOTCH1 pathway was later confirmed by the Dalla-Favera group to include CLL cells beyond genetic mutations, i.e., affecting also “bona fide” *NOTCH1*-wild type CLL cases. Increasing evidence, summarized in this review, suggests multiple mechanisms that ultimately result in chronic activation of a Notch-related transcription program involving either Notch-related genes (e.g., *FBXW7*, *SPEN*, *MED12*) or, more surprisingly, apparently unrelated cellular pathways (e.g., SF3B1, BCR, AKT). The emerging picture supports a model of multifaceted genetic pressure and environmental stimuli insisting on the NOTCH1 pathway; this may reflect a strategy where the neoplastic CLL cell is trying to exploit a physiological signal, normally required for B lymphocyte maturation and differentiation, for its own proliferative advantages [18].

Stimulation of the Notch pathway, along with the other main pathways induced by microenvironmental contacts through the BCR and integrins, may therefore be considered one of the hallmark signaling events distinguishing CLL cells from normal B cells that is necessary for CLL pathogenesis, progression, and Richter transformation [237,238]. One significant piece of evidence supporting this perspective is the typically low expression of the CD20 antigen in CLL, which has long been one of the main phenotypic CLL peculiarities recognized in CLL-specific scores [241], which occurs due to mechanisms related to the activation of the Notch pathway [128,134,175].

From a therapeutic standpoint, direct and effective therapeutic targeting of the NOTCH1 pathway has proven difficult [131] and even treatment with ibrutinib is not decisive [230,232]. Nevertheless, the landscape of therapeutic strategies targeting Notch signaling is rapidly expanding (reviewed in [242]) and may provide novel approaches, e.g., targeting DLL4-NOTCH1 interactions [146,243,244], or combinational therapies [245] to hit the Notch pathway in CLL.

## Data Availability

The data presented in this study are available in this article (and Supplementary Material).

## Supplementary Materials

The following supporting information can be downloaded at: https://www.mdpi.com/article/10.3390/cancers14122997/s1, File S1: Original blots.
