# Supplementary figures and images for: Multiple Mechanisms of NOTCH1 Activation in Chronic Lymphocytic Leukemia: NOTCH1 Mutations and Beyond
Source: Cancers (Basel). 2022 Jun 17;14(12):2997. doi: 10.3390/cancers14122997 (PMC9221163; doi:10.3390/cancers14122997)

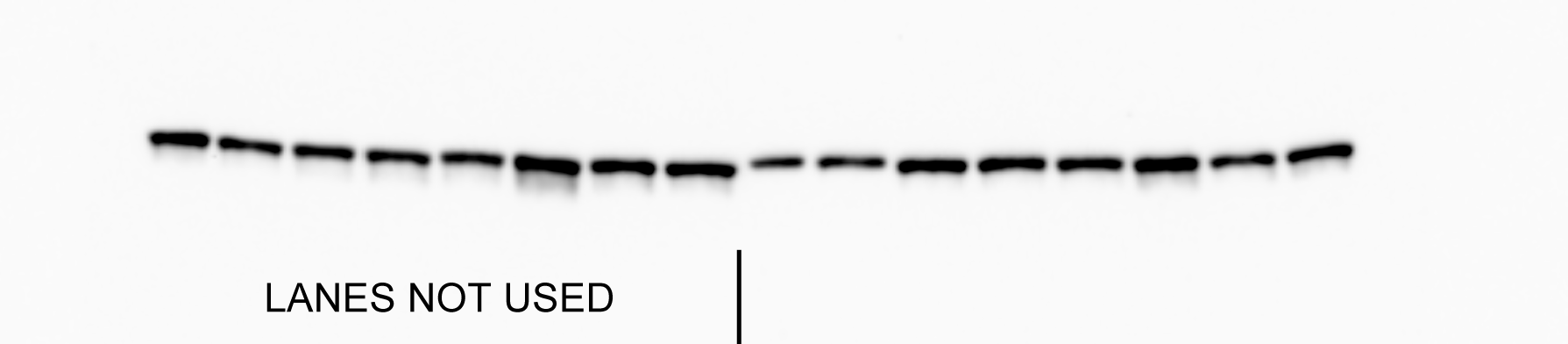

Supplement: Supplementary file 1 [file cancers-14-02997-s001.zip › Original Blots/Fig2_Actin.tif]

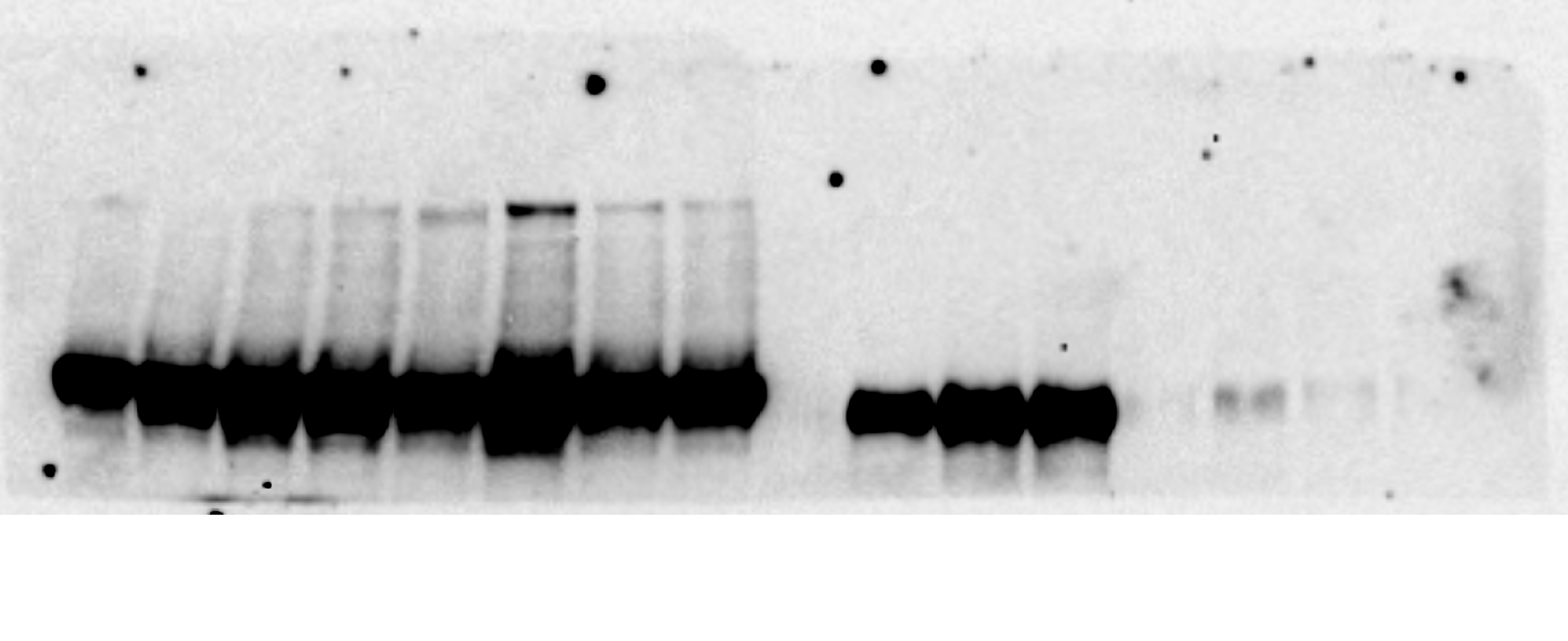

Supplement: Supplementary file 1 [file cancers-14-02997-s001.zip › Original Blots/Fig2_NICD_HighExp.tif]

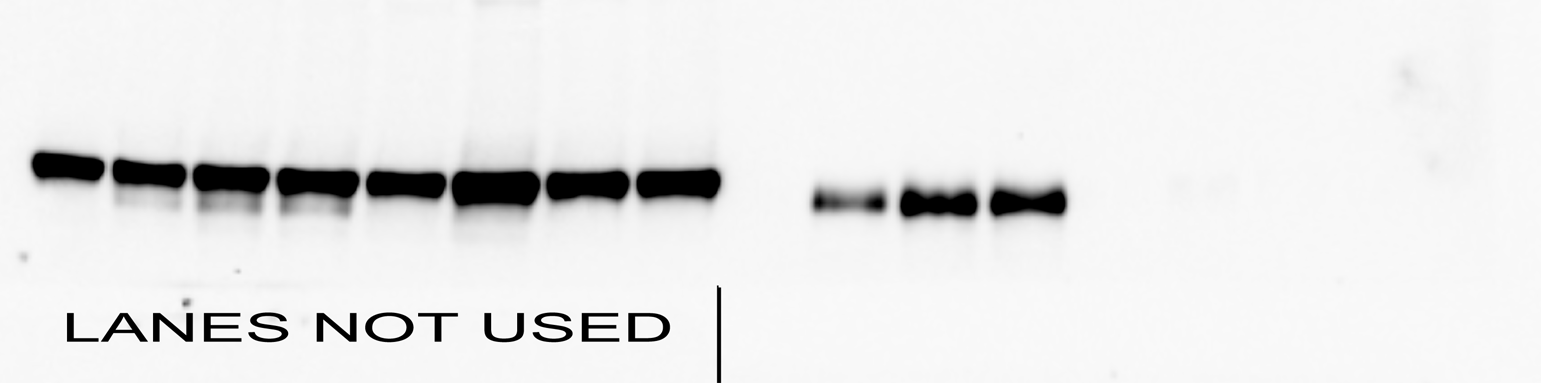

Supplement: Supplementary file 1 [file cancers-14-02997-s001.zip › Original Blots/Fig2_NICD_LowExp.tif]

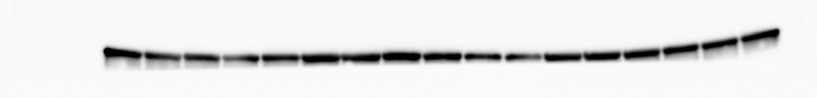

Supplement: Supplementary file 1 [file cancers-14-02997-s001.zip › Original Blots/Fig3_Actin.tif]

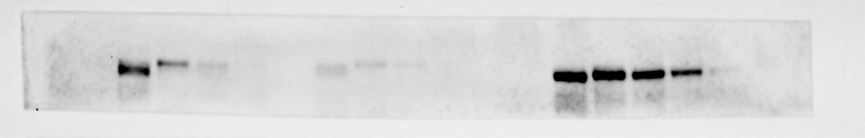

Supplement: Supplementary file 1 [file cancers-14-02997-s001.zip › Original Blots/Fig3_NICD.tif]

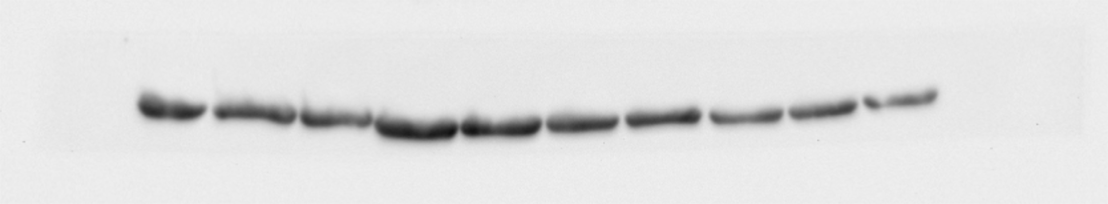

Supplement: Supplementary file 1 [file cancers-14-02997-s001.zip › Original Blots/Fig4_Actin.tif]

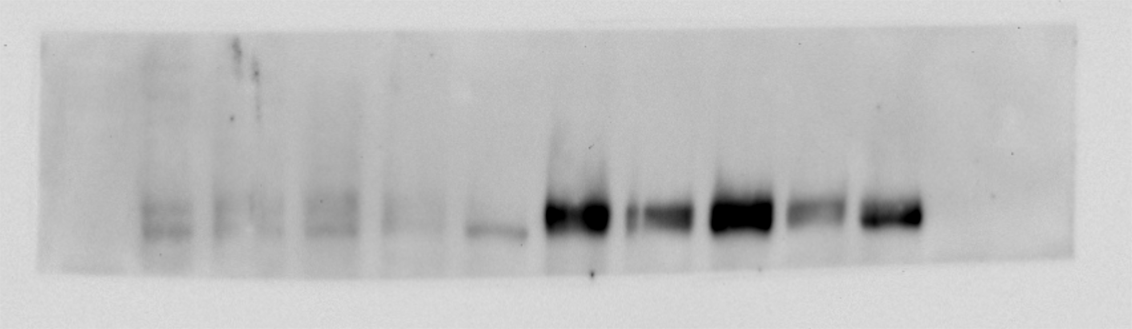

Supplement: Supplementary file 1 [file cancers-14-02997-s001.zip › Original Blots/Fig4_NICD_HIghExp.tif]

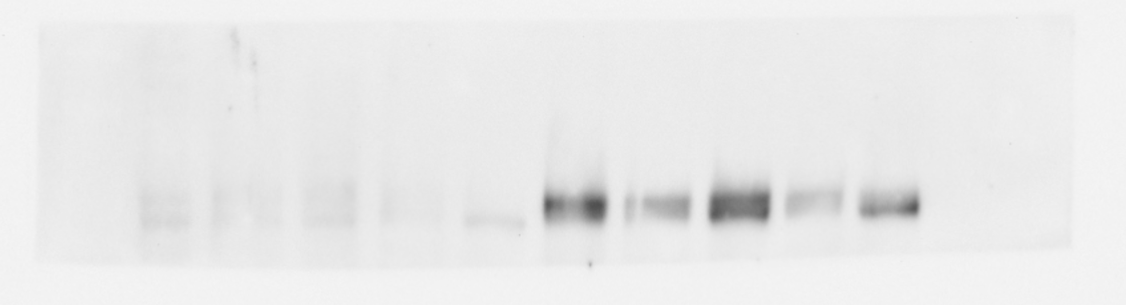

Supplement: Supplementary file 1 [file cancers-14-02997-s001.zip › Original Blots/Fig4_NICD_LowExp.tif]
